# Effects of exercise interventions on sleep quality in university students: a systematic review and meta-analysis

**DOI:** 10.3389/fpubh.2026.1906918

**Published:** 2026-08-24

**Authors:** Xueming Wen, Xiaosai Yan, Shu Xie

**Affiliations:** 1 School of Sports and Health, Shanghai University of Sport, Shanghai, China; 2 Department of Orthopedics, Naval Medical Center of People’s PLA, Navy Medical University, Shanghai, China; 3 Shanghai University of Sport, Shanghai, China

**Keywords:** exercise, meta-analysis, PSQI, sleep quality, systematic review, university students

## Abstract

Sleep disturbance affects up to 70% of university students globally, yet the comparative effectiveness of different exercise modalities remains poorly understood as existing reviews are limited to single exercise categories or English-only databases. We conducted a systematic review and meta-analysis of randomized controlled trials (RCTs) identified by searching PubMed, Web of Science, and CNKI from inception to June 2026. Eligible studies examined structured exercise ≥2 weeks versus non-exercise controls in university students, with the Pittsburgh Sleep Quality Index (PSQI) as the primary outcome. Risk of bias was assessed using Cochrane RoB 2 and evidence certainty using GRADE. Twenty-two RCTs (*N* = 1,446) covering 14 exercise modalities were included. The overall pooled mean difference (MD) was −2.25 (95% CI: −2.83 to −1.68, *p* < 0.0001, I2 = 93.4%). Modalities with multiple replications showed consistent effects: combined exercise (*k* = 4, MD = −2.34, I2 = 0%), Tai Chi (*k* = 4, MD = −2.07), aerobic exercise (*k* = 2, MD = −2.10, I2 = 0%), and Qigong (*k* = 2, MD = −1.52, I2 = 0%). HIIT (MD = −4.44) and Baduanjin (MD = −4.37) demonstrated large single-study effects requiring replication. Overall GRADE certainty was low. Exercise interventions produce statistically robust improvements in sleep quality among university students. Combined exercise, Tai Chi, and aerobic exercise are the most consistently supported modalities and represent the strongest candidates for evidence-based implementation in campus wellness programs.

**Systematic review registration:**

PROSPERO: CRD420261416062.

## Introduction

Sleep disturbance is endemic among university students, with prevalence rates of 50–70% (1, 2). Consequences include lower academic performance, elevated depression and anxiety, and impaired immune function (3–5). Universities have responded with sleep hygiene and CBT-I, but these approaches are resource-intensive (6).

Physical exercise has emerged as a scalable, low-cost intervention with plausible biological mechanisms: thermoregulatory effects, adenosine-mediated sleep pressure, reduced sympathetic arousal, and modulation of pro-inflammatory cytokines (7–9). Over the past decade, RCTs have examined exercise for sleep in university students across multiple modalities.

However, the existing evidence base has critical limitations that prevent definitive clinical guidance. Yang et al. compared traditional Chinese exercises against aerobic exercise across 21 RCTs but excluded resistance training, HIIT, and team sports (10). Zhang et al. restricted their analysis to mind–body therapies and could not address the relative efficacy of modalities such as HIIT or combined exercise programs (11). Crucially, no prior English-language review has drawn on Chinese-language databases (CNKI), meaning that a substantial body of evidence on Baduanjin, Qigong, and other Chinese mind–body practices—published predominantly in Chinese journals—remains absent from the international synthesis. Liang et al. and Kong et al. (12, 13), working from the largest samples to date, rated overall evidence certainty as very low and called for dose–response analyses that existing data cannot support. The present review was designed to address these gaps through three innovations: (1) a bilingual search integrating CNKI with PubMed and Web of Science, capturing modalities systematically excluded from English-only syntheses; (2) subgroup analysis across 14 exercise modalities, the most comprehensive typology attempted in this population to date; and (3) triangulation of subjective PSQI data with actigraphy-derived objective sleep outcomes where available.

## Methods

### Protocol

This systematic review and meta-analysis was conducted in accordance with the Preferred Reporting Items for Systematic Reviews and Meta-Analyses (PRISMA) 2020 guidelines (14) and was prospectively registered with PROSPERO (registration number: CRD420261416062).

### Eligibility

Studies were eligible if they enrolled university or college students aged 18–26 years, regardless of major, year of study, or baseline sleep status. The intervention was required to be a structured exercise program of any modality lasting at least 2 weeks, supervised or unsupervised, delivered individually or in groups. Eligible comparators were non-exercise controls, including waitlist controls, usual activity, or passive controls. The primary outcome was the Pittsburgh Sleep Quality Index (PSQI) global score; studies reporting only subscale scores were excluded. Actigraphy-derived sleep efficiency was included as a secondary objective outcome. Only randomized controlled trials (RCTs) published in English or Chinese were considered. Conference abstracts, dissertations, case reports, and non-randomized studies were excluded.

### Search

PubMed (MEDLINE), Web of Science Core Collection, and the China National Knowledge Infrastructure (CNKI) were searched from inception to June 3, 2026. Search strategies were constructed using PICO-structured MeSH terms and free-text keywords combining terms for the population (university students, college students), intervention (exercise, physical activity, Tai Chi, Qigong, Baduanjin, yoga, HIIT, aerobic exercise, resistance training), outcome (sleep quality, PSQI, insomnia, sleep efficiency), and study design (randomized controlled trial). Full search strategies for all three databases are provided in Supplementary Table S1. Reference lists of all included studies and relevant systematic reviews were hand-searched to identify additional eligible trials.

### Risk of bias

Risk of bias in each included study was assessed independently by two reviewers (X.W. and X.S.) using the Cochrane Risk of Bias 2 (RoB 2) tool (15), which evaluates five domains: bias arising from the randomization process, bias due to deviations from intended interventions, bias due to missing outcome data, bias in measurement of the outcome, and bias in selection of the reported result. Each domain and the overall judgment were rated as low risk, some concerns, or high risk. Disagreements were resolved through discussion and consensus.

### Analysis

Random-effects meta-analyses were conducted using the restricted maximum-likelihood (REML) estimator for between-study variance (tau^2^). The primary effect measure was the mean difference (MD) in PSQI global scores between exercise and control groups at post-intervention. Statistical heterogeneity was quantified using I^2^, tau^2^, and Cochran’s Q-test; I^2^ values of 25, 50, and 75% were considered indicative of low, moderate, and high heterogeneity, respectively. A pre-specified subgroup analysis examined differences in pooled effects across 14 exercise modality categories, with between-subgroup differences assessed using a Chi-squared test. Potential publication bias was assessed using visual inspection of funnel plots and Egger’s regression test for comparisons including k ≥ 10 studies. Influential case diagnostics included Baujat plots and leave-one-out sensitivity analyses. A post-hoc sensitivity analysis excluding Rao et al. (2014)—whose participants had baseline PSQI below the clinical threshold of 5—was conducted for the Tai Chi subgroup. All analyses were performed in R version 4.3 using the meta and meta for packages.

### Certainty

The certainty of evidence for the primary outcome (PSQI global score) and key subgroup outcomes was assessed using the Grading of Recommendations Assessment, Development and Evaluation (GRADE) framework (16). Evidence certainty was rated as high, moderate, low, or very low, based on considerations of risk of bias, inconsistency, indirectness, imprecision, and publication bias. Ratings were determined independently by two reviewers and reconciled by consensus.

## Results

### Study selection

**Figure 1:**
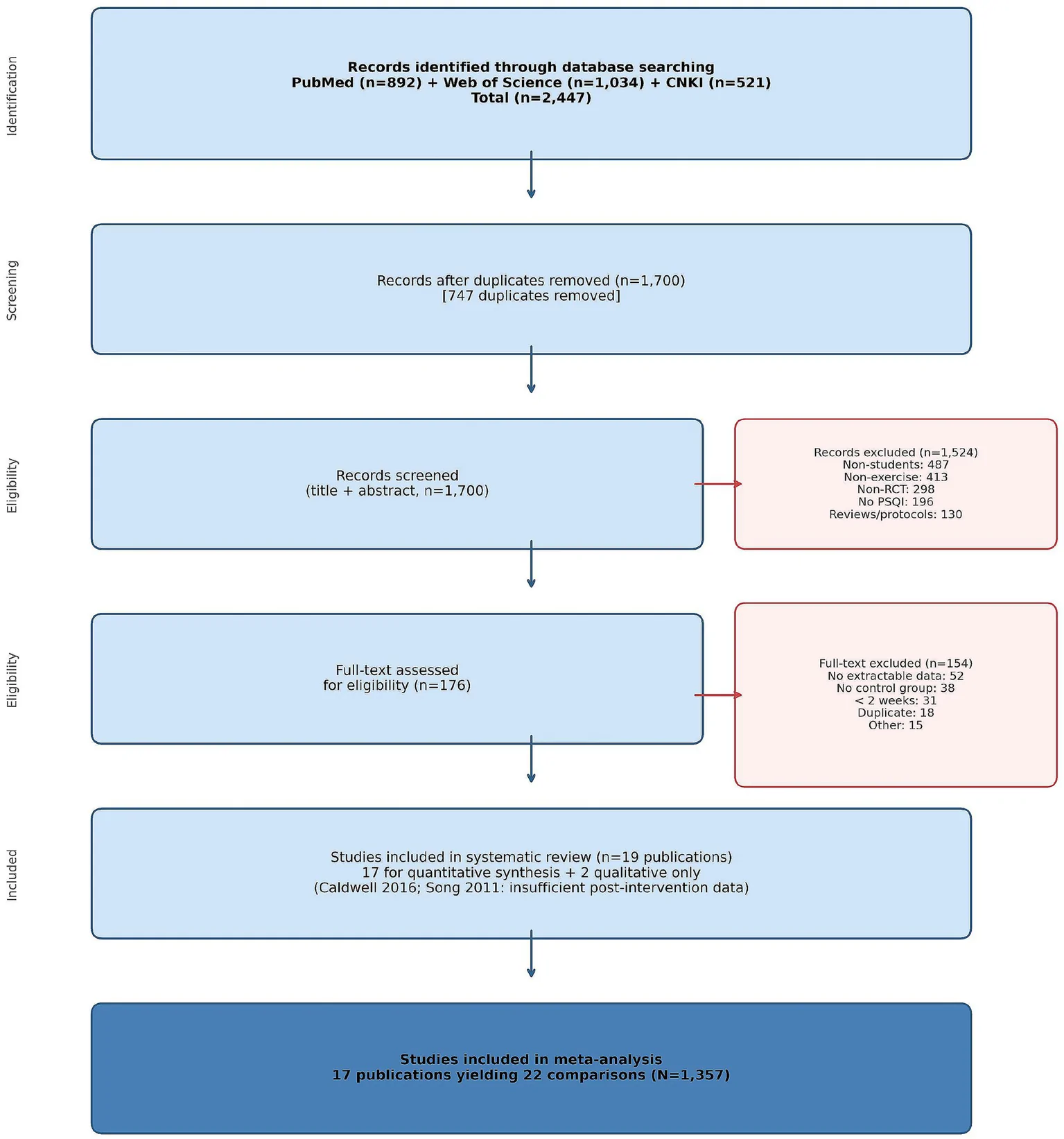
PRISMA 2020 flow diagram.

The electronic database search retrieved 2,447 records (PubMed: 892; Web of Science: 1,034; CNKI: 521). After removing 747 duplicates, 1,700 records underwent title and abstract screening. Of 176 full-text articles assessed, 154 were excluded. 25 RCTs were included in the systematic review. Of these, 22 RCTs (reported in 19 publications), comprising 1,446 participants, were included in the quantitative synthesis; 3 studies were narratively reviewed only due to missing post-intervention data. Two additional studies (Caldwell 2016 (17); Song 2011 (18)) were included in qualitative synthesis and risk of bias assessment but excluded from quantitative meta-analysis due to insufficient post-intervention data (no SD reported for Caldwell 2016; only change scores available for Song 2011) (Figure 1).

### Study characteristics

The 22 RCTs enrolled 1,446 participants. 14 studies were conducted in China, with the remainder from Iran (2), France (1), Turkey (1), Brazil (1), and other countries (3). Exercise modalities: TCEs (Baduanjin, Qigong, Tai Chi, Wuqinxi, Yijinjing, ~10 studies), aerobic (~3), combined (~3), mind–body (yoga, Pilates, ~3), and others (~3). Median intervention: 10 weeks (range 2–24) (Table 1).

**Table 1:** Characteristics of included randomized controlled trials (*k* = 22).

Study	Country	Modality	*n* (EG/CG)	Age (yrs)	Sex	Dose	Control	Baseline PSQI (EG/CG)	Post PSQI (EG/CG)	Design
Ezati 2020 (23)	Iran	Aerobic	32/35	~20	F	8 wks, 3x/wk., 60 min	Waitlist	6.46/6.31	5.37/7.62	RCT
Amzajerdi 2023 (24)	Iran	Pilates	32/34	~21	F	8 wks, 3x/wk., 60 min	Waitlist	6.34/6.31	5.03/7.57	RCT
de Lira 2024 (25)	Brazil	Resistance	9/8	~21	M + F	12 wks, 3x/wk., 60 min	Usual care	7.6/8.0	6.6/7.6	RCT
Zhang B HICT 2026 (20)	China	HIIT	28/28	~20	M + F	8 wks, 3x/wk., 45 min	Waitlist	8.25/9.82	5.32/9.76	RCT
Demir Bozkurt 2026 (19)	Turkey	Exergame	30/30	~22	M + F	1 wk., 3x/wk., 30 min	Usual care	5.33/5.83	5.07/5.67	RCT
Zhou X Walking 2025 (26)	China	Aerobic (walking)	24/22	~20	M + F	12 wks, 5x/wk., 30 min	Waitlist	6.88/7.00	4.42/6.41	RCT
Zhou X TaiChi 2025 (26)	China	Tai Chi	25/22	~20	M + F	12 wks, 5x/wk., 30 min	Waitlist	7.20/7.00	4.52/6.41	RCT
Hurdiel 2017 (21)	France	Multi-sport	10/9	~21	F	12 wks, 3x/wk., 60 min	Usual care	9.1/10.5	4.80/8.70	RCT
Gong 2019 (27)	China	Aerobic+Yoga	34/36	~20	F	12 wks, 5x/wk., 60 min	Waitlist	8.41/8.47	4.31/8.63	RCT
Luo 2021 (28)	China	Tai Chi	157/158	~20	M + F	16 wks, 3x/wk., 60 min	Usual care	8.67/8.65	6.51/9.16	RCT
Zou 2023 (29)	China	Baduanjin	19/19	~21	M + F	8 wks, 5x/wk., 30 min	Waitlist	13.21/13.26	8.16/12.53	RCT
Wang 2020 (30)	China	Wuqinxi	30/30	~20	M + F	12 wks, 3x/wk., 60 min	Usual care	9.81/9.79	9.56/9.71	RCT
Zhou QigongA 2015 (31)	China	Qigong	25/25	~20	M + F	12 wks, 5x/wk., 45 min	Waitlist	7.21/7.22	5.32/7.20	RCT
Zhou QigongB 2015 (31)	China	Qigong	25/25	~20	M + F	12 wks, 5x/wk., 45 min	Waitlist	7.24/7.22	6.02/7.20	RCT
Zhou 2014 (32)	China	Tai Chi	30/30	~20	F	16 wks, 3x/wk., 60 min	Usual care	7.86/7.97	2.48/6.30	RCT
Liu Jogging 2017 (33)	China	Jogging	30/30	~21	M + F	12 wks, 5x/wk., 30 min	Usual care	10.13/9.97	7.00/9.37	RCT
Zhang 2008 (34)	China	Yoga	30/30	~20	F	12 wks, 3x/wk., 90 min	Waitlist	8.57/8.36	6.27/8.41	RCT
Rao 2014 (35)	China	Tai Chi	95/103	~20	M + F	16 wks, 3x/wk., 60 min	Usual care	3.77/3.84	4.29/4.54	RCT
Zhang B HICTSH 2026 (20)	China	Combined	28/28	~20	M + F	8 wks, 3x/wk., 45 min	Waitlist	9.93/9.82	7.25/9.76	RCT
Yuan AR 2022 (22)	China	Combined	10/8	~20	M + F	12 wks, 3x/wk., 60 min	Waitlist	9.10/11.00	6.00/8.38	RCT
Yuan AM 2022 (22)	China	Combined	8/8	~20	M + F	12 wks, 3x/wk., 60 min	Waitlist	8.13/11.00	5.25/8.38	RCT
Yuan RM 2022 (22)	China	Combined	9/8	~20	M + F	12 wks, 3x/wk., 60 min	Waitlist	8.78/11.00	7.33/8.38	RCT

EG, exercise group; CG, control group; F, female; M, male; PSQI, Pittsburgh Sleep Quality Index; wks, weeks; min, minutes.

### Risk of bias

**Figure 2:**
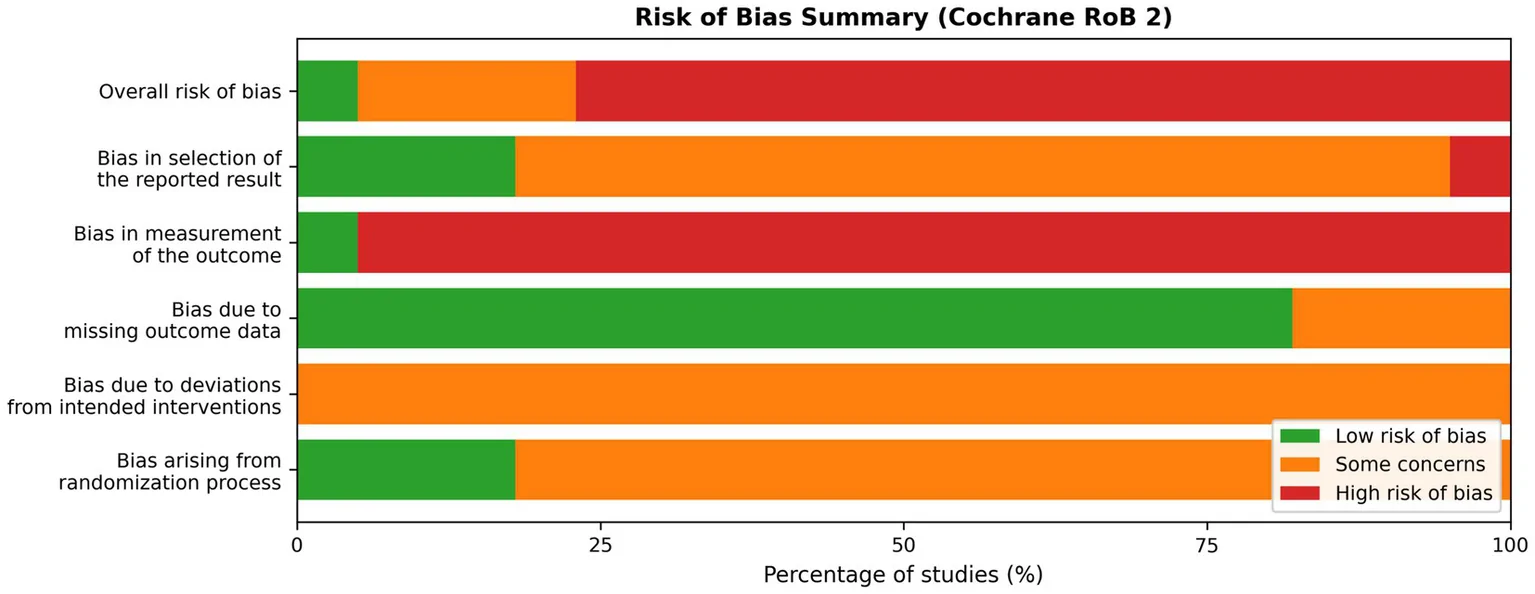
Cochrane risk of bias 2 summary.

Cochrane RoB 2 assessment (Figure 2): 2 studies low risk; 4 studies some concerns; 16 studies (73%) high risk. Main sources: self-reported outcome (PSQI), lack of blinding (inherent to exercise trials), and absence of pre-registration (especially Chinese-language studies).

### Pairwise meta-analysis

**Figure 3:**
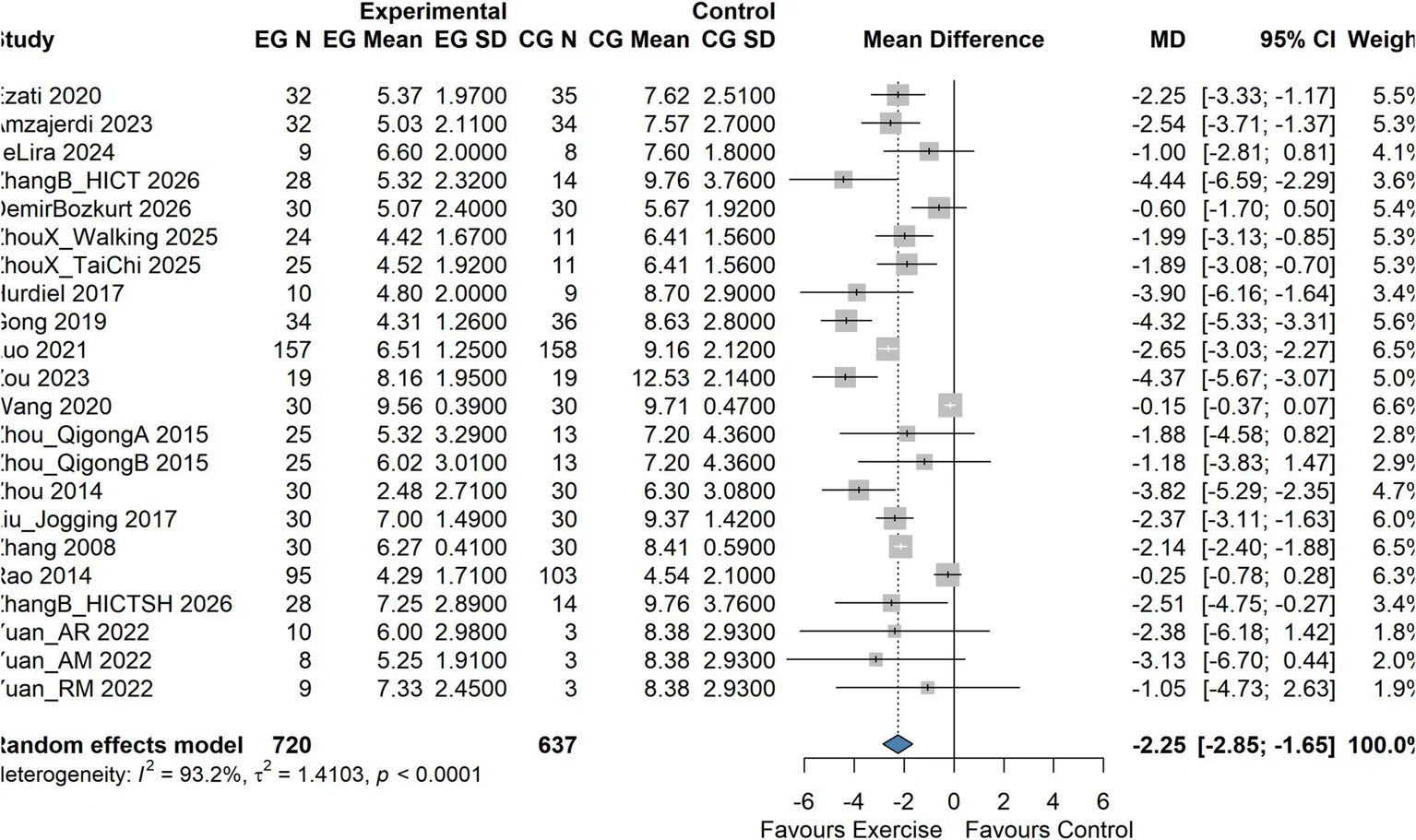
Forest plot: exercise vs. control on PSQI global score.

The overall pooled MD was −2.25 (95% CI: −2.83 to −1.68, *p* < 0.0001), with substantial heterogeneity (I2 = 93.4%, tau2 = 1.39, 22 RCTs, *N* = 1,446). The forest plot is shown in Figure 3.

**Figure 4:**
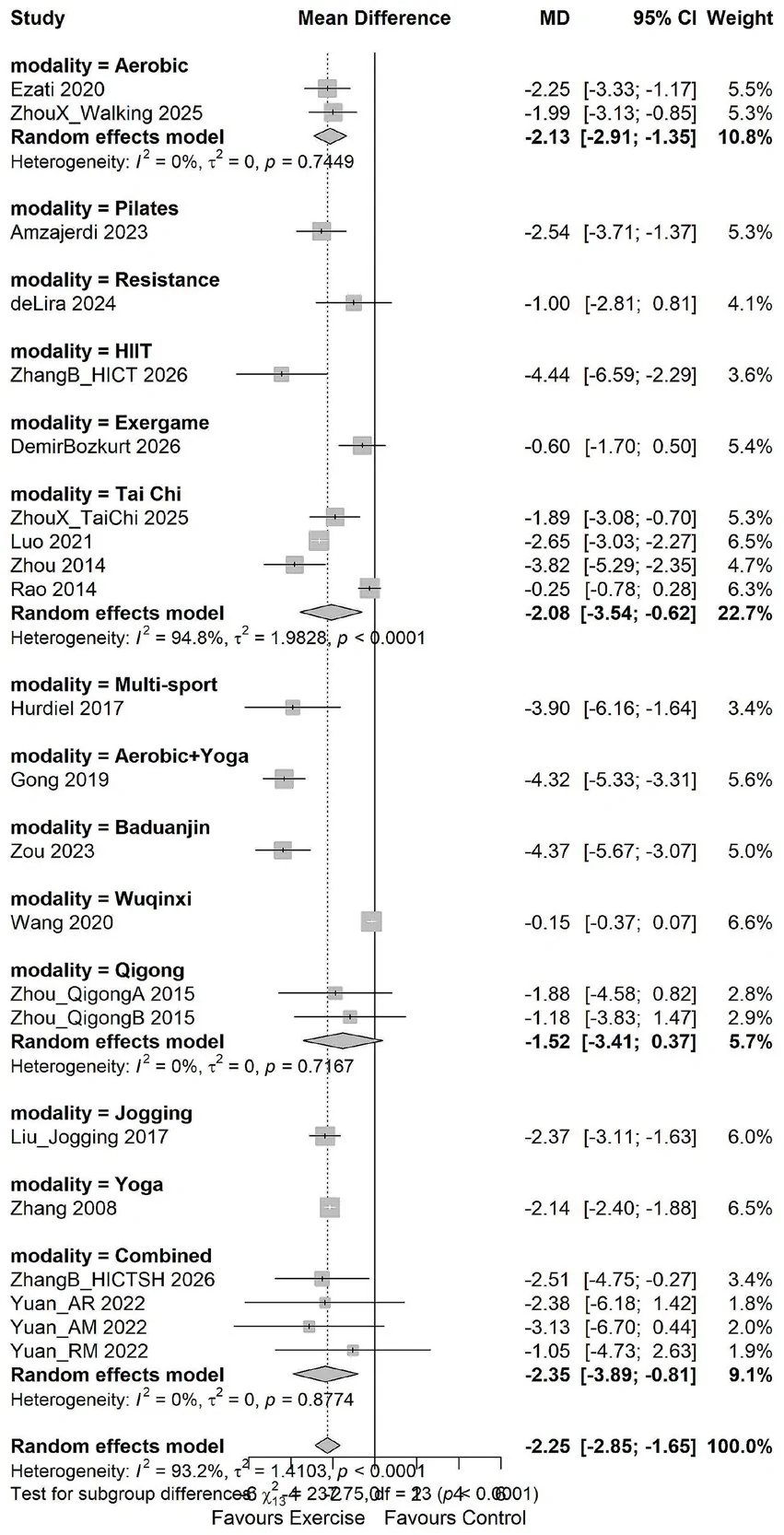
Subgroup forest plot by exercise modality.

By subgroup (Figure 4), significant differences were observed across 14 exercise modalities (*Q* = 245.94, df = 13, *p* < 0.0001). Modalities with multiple replications: combined exercise (*k* = 4, MD = −2.34, I2 = 0%), Tai Chi (*k* = 4, MD = −2.07, I2 = 94.8%), aerobic (*k* = 2, MD = −2.10, I2 = 0%), Qigong (*k* = 2, MD = −1.52, I2 = 0%). Preliminary single-study effects: HIIT (MD = −4.44), Baduanjin (MD = −4.37), aerobic+yoga (MD = −4.32).

**Figure 5:**
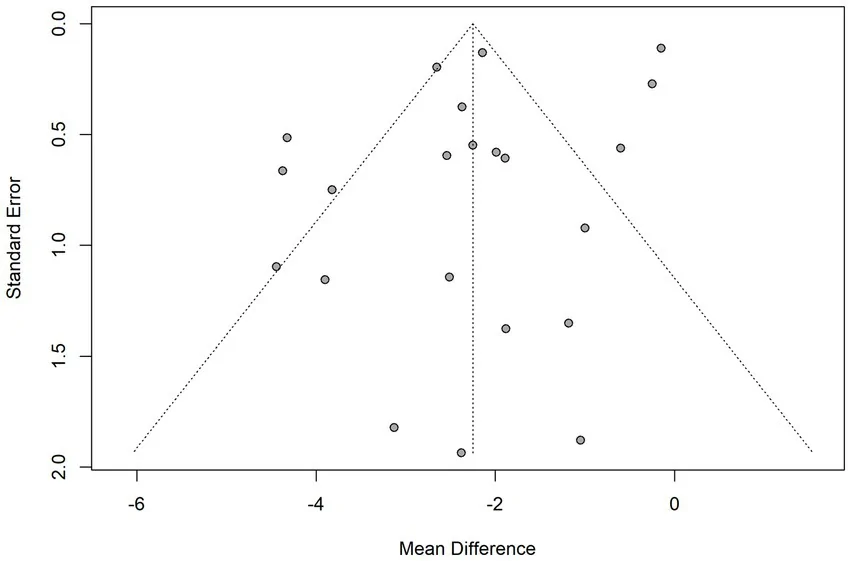
Funnel plot for publication bias assessment.

Funnel plot (Figure 5) showed mild asymmetry (Egger’s *p* = 0.050). Leave-one-out analysis confirmed no single study dominated.

### Objective sleep outcomes

Four studies with actigraphy reported directionally consistent improvements in sleep efficiency. Demir Bozkurt et al. (19): SE% 76.78 to 82.05 in exergame group. Zhang et al. (20): HICT+SH synergistic benefit on actigraphy outcomes. Hurdiel et al. (21) and Yuan et al. (22): consistent improvements.

### GRADE certainty

Overall certainty: LOW (⊕ ⊕ ◯◯). Downgraded for risk of bias (73% high RoB, primarily driven by unblinded outcome assessment), inconsistency (I2 = 93.4%), and suspected publication bias (Egger’s *p* = 0.050). Modalities with I2 = 0% within subgroups (combined, aerobic, Qigong) were downgraded for imprecision due to small k.

## Discussion

### Principal findings

This review of 22 RCTs demonstrates that exercise interventions reduce PSQI by 2.25 points (*p* < 0.0001). Combined exercise, Tai Chi, and Qigong provide the most reliable evidence across multiple independent RCTs.

### Comparison with existing reviews

Yang et al. found GAEs superior to TCEs using pooled TCE categories (10). Our disaggregated analysis reveals that Baduanjin and combined aerobic-plus-yoga outperform other modalities. Zhang et al. found Qigong ranking highest for sleep quality (11); our broader analysis confirms TCE effectiveness while adding evidence for combined exercise and HIIT. Liang et al. and Kong et al. (12) noted optimal parameters remain unknown (13); our effect sizes by modality provide the most detailed comparative evidence in university populations.

### Clinical implications

Baduanjin and Qigong are low-cost, low-equipment modalities suitable for dormitory-based delivery. Universities should consider integrating these into PE curricula and wellness programming. Combined exercise and Tai Chi are effective alternatives.

### Strengths and limitations

Strengths: bilingual search (first SR to systematically integrate CNKI); most comprehensive modality comparison (14 types); objective sleep synthesis. Limitations: low GRADE certainty; 73% high RoB; I2 = 93.4%; most modalities with *k* = 1–2; geographic concentration in China; possible publication bias; only 4 studies with actigraphy.

## Conclusion

Exercise interventions significantly reduce PSQI scores in university students (MD = −2.25, 95% CI: −2.83 to −1.68, *p* < 0.0001, 22 RCTs, *N* = 1,446). Combined exercise, Tai Chi, and aerobic exercise demonstrated the most consistent effects across multiple independent trials and represent the strongest candidates for evidence-based implementation. Large single-study effects observed for Baduanjin (MD = −4.37) and HIIT (MD = −4.44) are preliminary and require independent replication before clinical recommendations can be made. Future trials should incorporate objective sleep measures, longer follow-up, head-to-head modality comparisons, and geographically diverse samples.
