# Supplementary material for: Effects of exercise interventions on sleep quality in university students: a systematic review and meta-analysis
Source: Front Public Health. 2026 Aug 24;14:1906918. doi: 10.3389/fpubh.2026.1906918 (PMC13548498; doi:10.3389/fpubh.2026.1906918)
Supplement: Supplementary file 1 [file Supplementary_file_1.docx]

**PRISMA 2020 Checklist**

Manuscript: Effects of Exercise Interventions on Sleep Quality in University Students: A Systematic Review and Meta-Analysis

Publication version: manuscript locations are indicated by section headings and named supplementary items; no manuscript page numbers are used.

| **Section** | **Item** | **Checklist item** | Location in manuscript |
| --- | --- | --- | --- |
| Title | 1 | Identify the report as a systematic review | Title |
| Abstract | 2 | Structured summary including background, objectives, data sources, eligibility criteria, results, conclusions | Abstract |
| Introduction | 3a | Describe the rationale for the review | Introduction |
| Introduction | 3b | Provide an explicit statement of the objectives | Introduction |
| Methods – Eligibility | 4 | Specify inclusion and exclusion criteria | Methods - Eligibility Criteria |
| Methods – Information sources | 5 | Describe all information sources searched and date of last search | Methods - Information Sources; Supplementary Table S1 |
| Methods – Search | 6 | Present full search strategy for at least one database | Supplementary Table S1 |
| Methods – Selection | 7 | Describe screening and selection process | Methods - Study Selection and Data Extraction |
| Methods – Data collection | 8 | Describe method of data extraction | Methods - Study Selection and Data Extraction |
| Methods – Data items | 9 | List and define outcomes and other variables | Methods - Study Selection and Data Extraction |
| Methods – RoB | 10 | Describe method for assessing RoB in included studies | Methods - Risk of Bias; Supplementary Table S3 |
| Methods – Synthesis | 11 | Describe methods for statistical synthesis | Methods - Statistical Analysis |
| Methods – RoB across studies | 12 | Describe any methods to assess publication bias | Methods - Statistical Analysis |
| Results – Selection | 13 | Give numbers of identified, screened, eligible, and included studies (PRISMA flow diagram) | Results - Study Selection; Figure 1 |
| Results – Characteristics | 14 | Present characteristics of included studies | Results - Study Characteristics; Table 1 |
| Results – RoB | 15 | Present RoB for each included study | Results - Risk of Bias; Figure 2; Supplementary Table S3 |
| Results – Synthesis | 16 | Present results of all statistical analyses | Results - Pairwise Meta-Analysis and Exploratory Modality Analysis; Figures 3-5; Supplementary Table S2 |
| Results – RoB across studies | 17 | Present results of publication bias assessment | Results - Publication Bias; Figure 5 |
| Discussion – Summary | 18 | Summarize main findings | Discussion |
| Discussion – Limitations | 19 | Discuss limitations | Discussion - Strengths and Limitations |
| Discussion – Conclusions | 20 | Provide general interpretation of results | Conclusion |
| Other – Registration | 21 | Provide registration number and name of registry | Methods - Protocol and Registration; PROSPERO CRD420261416062 |
| Other – Protocol | 22 | Indicate where protocol can be accessed | PROSPERO record: CRD420261416062 |
| Other – Support | 23 | Describe sources of financial support | Declarations - Funding |
| Other – Competing interests | 24 | Declare competing interests | Declarations - Conflict of Interest |
| Other – Availability | 25 | Report availability of data, code, and other materials | Declarations - Data Availability; Supplementary Data and R Code archive |
| Other – AI | 26 | Report use of AI tools | Declarations - Generative AI and AI-assisted technologies |
