# Supplementary material for: Effects of exercise interventions on sleep quality in university students: a systematic review and meta-analysis
Source: Front Public Health. 2026 Aug 24;14:1906918. doi: 10.3389/fpubh.2026.1906918 (PMC13548498; doi:10.3389/fpubh.2026.1906918)
Supplement: Supplementary file 2 [file Table_1.DOCX]

**Supplementary Table S1. Full Electronic Search Strategies**

Search date: June 3, 2026. All databases searched from inception.

**PubMed (MEDLINE)**

| **Line** | **Search term** |
| --- | --- |
| #1 | ("university students"[MeSH] OR "college students"[MeSH] OR "students"[MeSH] OR "undergraduate"[tiab] OR "university student*"[tiab] OR "college student*"[tiab]) |
| #2 | ("exercise"[MeSH] OR "physical activity"[MeSH] OR "exercise therapy"[MeSH] OR "tai ji"[MeSH] OR "qigong"[MeSH] OR "yoga"[MeSH] OR "aerobic exercise"[tiab] OR "resistance training"[tiab] OR "HIIT"[tiab] OR "high intensity interval training"[tiab] OR "Baduanjin" OR "Yijinjing"[tiab] OR "Tai Chi"[tiab] OR "Qigong"[tiab] OR "combined exercise"[tiab] OR "exergame*"[tiab] OR "walking"[tiab] OR "jogging"[tiab] OR "Pilates"[tiab]) |
| #3 | ("sleep quality"[MeSH] OR "sleep"[MeSH] OR "sleep disorders"[MeSH] OR "sleep quality"[tiab] OR "sleep disturbance*"[tiab] OR "Pittsburgh Sleep Quality Index"[tiab] OR "PSQI"[tiab] OR "insomnia"[tiab] OR "sleep efficiency"[tiab]) |
| #4 | ("randomized controlled trial"[pt] OR "RCT"[tiab] OR "random*"[tiab]) |
| #5 | #1 AND #2 AND #3 AND #4 |

**Web of Science Core Collection**

| **Line** | **Search term** |
| --- | --- |
| #1 | TS=("university student*" OR "college student*" OR "undergraduate" OR "higher education student*") |
| #2 | TS=("exercise" OR "physical activity" OR "aerobic exercise" OR "resistance training" OR "Tai Chi" OR "Qigong" OR "Baduanjin" OR "Yijinjing" OR "yoga" OR "HIIT" OR "high-intensity interval training" OR "exergame*" OR "Pilates" OR "jogging" OR "walking" OR "combined exercise" OR "Wuqinxi" OR "mind-body exercise") |
| #3 | TS=("sleep quality" OR "sleep disturbance*" OR "PSQI" OR "Pittsburgh Sleep Quality Index" OR "insomnia" OR "sleep disorder*" OR "sleep efficiency" OR "actigraphy") |
| #4 | TS=("randomized controlled trial" OR "RCT" OR "randomised" OR "random assignment") |
| #5 | #1 AND #2 AND #3 AND #4 |

**CNKI (China National Knowledge Infrastructure)**

| **检索项** | **检索式** |
| --- | --- |
| #1 | 主题=("大学生" OR "高校学生" OR "本科生" OR "在校学生") |
| #2 | 主题=("运动干预" OR "体育锻炼" OR "有氧运动" OR "太极拳" OR "气功" OR "八段锦" OR "易筋经" OR "五禽戏" OR "瑜伽" OR "慢跑" OR "健步走" OR "抗阻训练" OR "高强度间歇训练" OR "HIIT" OR "组合运动" OR "体感游戏" OR "普拉提") |
| #3 | 主题=("睡眠质量" OR "匹兹堡睡眠质量指数" OR "PSQI" OR "失眠" OR "睡眠障碍" OR "睡眠效率") |
| #4 | 主题=("随机对照试验" OR "RCT" OR "随机分组" OR "随机对照") |
| #5 | #1 AND #2 AND #3 AND #4 |

Note: Search terms were adapted for each database syntax. MeSH terms used for PubMed; topic search (TS) used for Web of Science; subject search used for CNKI. Hand-searching of reference lists of included studies and relevant systematic reviews was also performed.
