# Supplementary material for: Effects of exercise interventions on sleep quality in university students: a systematic review and meta-analysis
Source: Front Public Health. 2026 Aug 24;14:1906918. doi: 10.3389/fpubh.2026.1906918 (PMC13548498; doi:10.3389/fpubh.2026.1906918)
Supplement: Supplementary file 3 [file Table_2.DOCX]

# Supplementary Table S2. Exploratory univariable meta-regressions

Each moderator was examined separately using a random-effects REML model with Hartung-Knapp inference. Negative coefficients indicate a larger reduction in post-intervention PSQI as the moderator increases. These analyses were post hoc and should be interpreted as exploratory.

| Moderator | k | β | SE | 95% CI | p | Residual I² | Residual τ² |
| --- | --- | --- | --- | --- | --- | --- | --- |
| Baseline PSQI | 13 | -0.265 | 0.238 | -0.789 to 0.258 | 0.288 | 93.2% | 1.492 |
| Intervention duration (weeks) | 13 | 0.084 | 0.049 | -0.025 to 0.192 | 0.117 | 90.0% | 1.149 |
| Sessions per week | 13 | 0.256 | 0.375 | -0.569 to 1.081 | 0.509 | 91.4% | 1.501 |
| Minutes per session | 13 | 0.011 | 0.022 | -0.037 to 0.058 | 0.635 | 91.2% | 1.582 |
| Exercise minutes per week | 13 | 0.010 | 0.007 | -0.005 to 0.025 | 0.174 | 91.8% | 1.307 |
| Total intervention minutes | 13 | 0.000 | 0.000 | 0.000 to 0.001 | 0.042 | 89.0% | 0.902 |

PSQI, Pittsburgh Sleep Quality Index; REML, restricted maximum likelihood; CI, confidence interval.
