# Supplementary material for: Effects of exercise interventions on sleep quality in university students: a systematic review and meta-analysis
Source: Front Public Health. 2026 Aug 24;14:1906918. doi: 10.3389/fpubh.2026.1906918 (PMC13548498; doi:10.3389/fpubh.2026.1906918)
Supplement: Supplementary file 4 [file Table_3.DOCX]

# Supplementary Tables S3-S4. Risk of Bias and GRADE Evidence Profile

## Supplementary Table S3. Study-level overall RoB 2 judgments

The main manuscript Figure 2 summarizes the 13 studies contributing to the primary meta-analysis. This table also reports the three narrative-only studies. Judgments were made independently by two reviewers and reconciled by discussion.

| Study | Synthesis | Overall RoB 2 | Main basis |
| --- | --- | --- | --- |
| de Lira 2024 | Primary meta-analysis | High | Self-reported outcome and limited reporting of randomization and selective reporting |
| ZhangB 2026 | Primary meta-analysis | Some concerns | Deviations from intended intervention and self-reported primary outcome |
| ZhouX 2025 | Primary meta-analysis | Some concerns | Deviations from intended intervention and self-reported primary outcome |
| Hurdiel 2017 | Primary meta-analysis | High | Self-reported outcome and limited reporting of randomization and selective reporting |
| Gong 2019 | Primary meta-analysis | High | Self-reported outcome and limited reporting of randomization and selective reporting |
| Luo 2021 | Primary meta-analysis | Some concerns | Deviations from intended intervention and self-reported primary outcome |
| Zou 2023 | Primary meta-analysis | High | Self-reported outcome and limited reporting of randomization and selective reporting |
| Wang 2020 | Primary meta-analysis | High | Self-reported outcome and limited reporting of randomization and selective reporting |
| Zhou Qigong 2015 | Primary meta-analysis | High | Self-reported outcome and limited reporting of randomization and selective reporting |
| Zhou Tai Chi 2014 | Primary meta-analysis | High | Self-reported outcome and limited reporting of randomization and selective reporting |
| Liu Jogging 2017 | Primary meta-analysis | High | Self-reported outcome and limited reporting of randomization and selective reporting |
| Zhang Yoga 2008 | Primary meta-analysis | High | Self-reported outcome and limited reporting of randomization and selective reporting |
| Yuan 2022 | Primary meta-analysis | High | Self-reported outcome and limited reporting of randomization and selective reporting |
| Caldwell 2016 | Narrative synthesis | Low | Randomized design with registered trial and objective outcome reporting |
| Song 2011 | Narrative synthesis | High | Incomplete compatible outcome reporting and self-reported outcome |
| Papp 2019 | Narrative synthesis | Some concerns | Pilot trial with incomplete compatible outcome reporting for this synthesis |

## Supplementary Table S4. GRADE evidence profile for the primary PSQI outcome

| Outcome | Studies | Participants | Risk of bias | Inconsistency | Indirectness | Imprecision | Other considerations | Effect | Certainty |
| --- | --- | --- | --- | --- | --- | --- | --- | --- | --- |
| Post-intervention PSQI global score | 13 RCTs | 964 | Serious (-1) | Serious (-1) | Not serious | Not serious | Possible small-study effects; Egger's regression was inconclusive and no additional downgrade was applied | MD 2.55 points lower (95% CI 3.35 lower to 1.74 lower) | Low |

GRADE began at high certainty for randomized evidence and was downgraded one level for risk of bias and one level for inconsistency. PSQI, Pittsburgh Sleep Quality Index; MD, mean difference; CI, confidence interval; RCT, randomized controlled trial.
